# The Involvement of Cx43 in JNK1/2-Mediated Endothelial Mechanotransduction and Human Plaque Progression

**DOI:** 10.3390/ijms24021174

**Published:** 2023-01-07

**Authors:** Miyuki Tauchi, Kensuke Oshita, Katharina Urschel, Roman Furtmair, Constanze Kühn, Florian M. Stumpfe, Balazs Botos, Stephan Achenbach, Barbara Dietel

**Affiliations:** 1 Department of Cardiology and Angiology, Erlangen University Hospital, Friedrich-Alexander University Erlangen-Nürnberg (FAU), 91054 Erlangen, Germany; 2 Cognitive and Molecular Research Institute of Brain Diseases, Kurume University, Kurume 830-0011, Japan; 3 Department of Anesthesiology, School of Medicine, Kurume University, Kurume 830-0011, Japan; 4 Department of Vascular Surgery, Hospital of Nürnberg-Süd, 90471 Nürnberg, Germany

**Keywords:** atherogenesis, shear stress, mechanotransduction, MAPK, endothelial dysfunction, gap junctions

## Abstract

Atherosclerotic lesions preferentially develop at bifurcations, characterized by non-uniform shear stress (SS). The aim of this study was to investigate SS-induced endothelial activation, focusing on stress-regulated mitogen-activated protein kinases (MAPK) and downstream signaling, and its relation to gap junction proteins, Connexins (Cxs). Human umbilical vein endothelial cells were exposed to flow (“mechanical stimulation”) and stimulated with TNF-α (“inflammatory stimulation”). Phosphorylated levels of MAPKs (c-Jun N-terminal kinase (JNK1/2), extracellular signal-regulated kinase (ERK), and p38 kinase (p38K)) were quantified by flow cytometry, showing the activation of JNK1/2 and ERK. THP-1 cell adhesion under non-uniform SS was suppressed by the inhibition of JNK1/2, not of ERK. Immunofluorescence staining and quantitative real-time PCR demonstrated an induction of c-Jun and c-Fos and of Cx43 in endothelial cells by non-uniform SS, and the latter was abolished by JNK1/2 inhibition. Furthermore, plaque inflammation was analyzed in human carotid plaques (*n* = 40) using immunohistochemistry and quanti-gene RNA-assays, revealing elevated Cx43^+^ cell counts in vulnerable compared to stable plaques. Cx43^+^ cell burden in the plaque shoulder correlated with intraplaque neovascularization and lipid core size, while an inverse correlation was observed with fibrous cap thickness. Our results constitute the first report that JNK1/2 mediates Cx43 mechanoinduction in endothelial cells by atheroprone shear stress and that Cx43 is expressed in human carotid plaques. The correlation of Cx43^+^ cell counts with markers of plaque vulnerability implies its contribution to plaque progression.

## 1. Introduction

Atherosclerosis is a chronic inflammatory disease of the arterial wall, characterized by the deposition of excess cholesterol, forming plaque in the intima of the arterial wall. It may trigger diseases that are leading causes of death, such as ischemic heart disease and stroke [1]. Patients with intracranial atherosclerotic stenosis of ≥70% had significantly higher risk of recurrent stroke compared with those with <70% stenosis [2], demonstrating the importance of intervention. Revascularization by means of angioplasty, such as percutaneous cardiovascular interventions with or without stent, as well as endarterectomy, is performed effectively to treat stenosis [3]; however, the relatively high restenosis rate is a limiting factor of effectiveness of these therapies [4,5].

Apart from pronounced immune responses, atherogenesis is also substantially influenced by local hemodynamics, such as hydrostatic pressure, cyclic stretch and shear stress (SS) [6,7,8,9]. In clinical practice, flow-mediated dilation is one of the most important tools to assess endothelial function [10]. While straight regions of the vasculature are barely affected by atherosclerotic plaques, bends or bifurcations are prone to plaque development, due to local flow patterns [11]. It is well established that the frictional force mediated by blood flow, so called shear stress (SS), acts on the inner layer of the vessel wall, affecting the activation of the endothelial cell (EC) monolayer [12]. Analyzing flow velocity profiles and SS in autopsy specimens, Zarins et al. showed in 1983 that low or oscillatory SS and a turbulent flow pattern, which was present in carotid bifurcations, predisposed these vessel regions for plaque development [13]. Vessel segments with moderate to high SS, which showed unidirectional flow, indeed remained spared from atherosclerotic plaque formation. The local blood flow pattern is sensed by ECs and transduced into biochemical signaling, which influences endothelial phenotype and function [14,15]. At atheroresistant regions of the vessel wall, ECs exert a quiescent non-activated phenotype. In contrast, ECs at atheroprone regions show increased endothelial dysfunction, resulting in enhanced permeability, proliferation and adhesiveness [14,15,16].

Several signaling pathways are involved in SS-induced endothelial mechanotransduction [16,17,18,19,20]. One of the most thoroughly characterized mechanosensing pathways is the complex containing platelet endothelial cell adhesion molecule-1 (PECAM1), vascular endothelial (VE)-Cadherin and vascular endothelial growth factor receptor 2 (VEGFR2) [20].

Apart from this complex, mitogen-activated protein kinases (MAPKs), comprising extracellular signal–regulated kinases (ERK), c-Jun N-terminal kinases (JNK) and p38 kinases (p38K), have been demonstrated for decades to be involved in SS-induced endothelial signaling [17,18,19]. In addition to their susceptibility to SS, ERK, JNK and p38K have also been reported to be TNF-α responsive [21,22]. While prior studies revealed that the mechanoactivation of MAP kinases is both force- and time-dependent [23] and varies from transient to persistent activation, a detailed role of these stress-responsive kinases in SS-induced endothelial activation still needs to be elucidated. Downstream pathways which are activated by MAP kinases include a wide range of signaling molecules, including transcription factors, cytokines, as well as molecules involved in inflammation, apoptosis, cell differentiation and intercellular communication and transport, such as gap junction molecules [24,25,26,27]. Gap junctions are composed of connexins, which are capable to oligomerize to hemichannels, connecting adjacent cells. Connexins, such as Cx43 and Cx40, on which we concentrated in our study, are expressed in vascular endothelial cells [28] and gain increasing attention in atherosclerosis research, as they were reported to be involved in the regulation of endothelial permeability and vascular inflammation [29]. Cx43 is expressed in smooth muscle cells as well, and upregulated at intimal thickening of early atherogenic plaque [30] and the lesion of post-endovcascular intervention [31,32]; therefore, increased expression of Cx43 is considered to play a pivotal role in restenosis of patients who underwent endovascular intervention.

A better understanding of the pathways involved in endothelial mechanoactivation might contribute to develop new strategies to target endothelial dysfunction. Thus, the aim of our study was to test the hypothesis that the atherogenic SS-induced MAPK pathway drives endothelial dysfunction by modulation of gap junction molecule expression. For this purpose, we investigated how different SS patterns affect mechanotransduction in human ECs, with regard to the involvement of MAPKs, focusing on ERK, JNK1/2, and p38K. Respective downstream signaling pathways were analyzed, including gap junction proteins, cytokines and proapoptotic signaling molecules, regulated by MAPKs [24,33,34].

## 2. Results

### 2.1. JNK1/2 Was Activated in HUVECs by Inflammatory and Mechanical Stimuli

**Figure 1:**
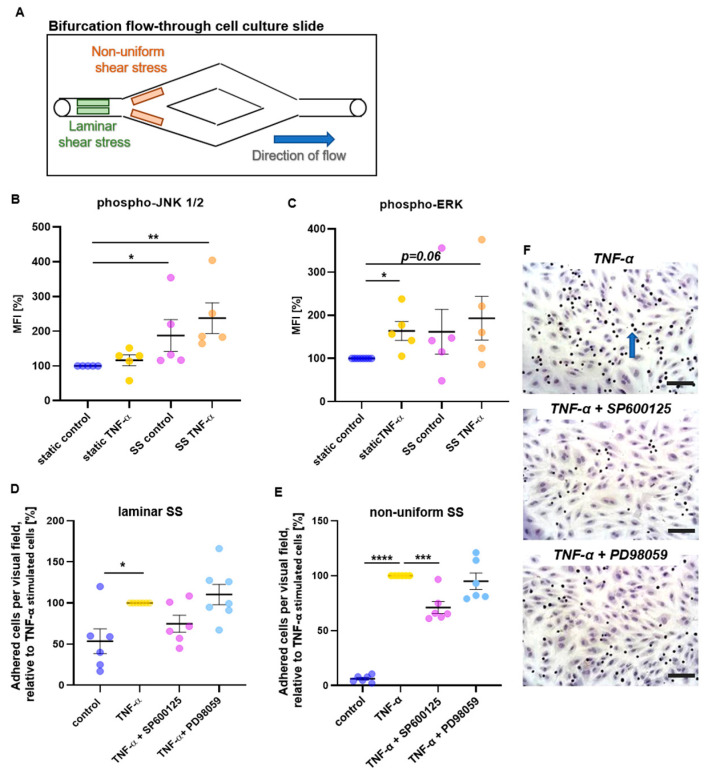
MAP kinase JNK1/2 is involved in TNF-α-boosted endothelial mechanoactivation. (**A**–**C**) Dynamic flow experiments were performed using bifurcation flow-through cell culture slides that create regions with laminar and non-uniform SS (**A**). Mean fluorescence intensity (MFI) of phosphorylated JNK1/2 (**B**) and phosphorylated ERK (**C**) was assessed by flow cytometric measurements and compared with static control. Phosphorylation of JNK1/2 and ERK was increased by the mechanical and inflammatory stimulation. Data are presented as mean ± SEM, *n* = 5, analyzed by Kruskal–Wallis test followed by Dunn’s multiple comparison (**D**–**F**). Same settings of the above-described dynamic flow experiments were used with additional application of kinase inhibitors (JNK1/2 inhibitor SP600125, ERK inhibitor PD98059) followed by 1 h of perfusion with THP-1 monocytes. Cells were stained and microscopic images in regions of laminar SS (6 per slide) and non-uniform SS (8 per slide) were obtained. Mean numbers of adherent THP-1 cells per visual field (0.25 mm^2^) were assessed in laminar (**D**) and non-uniform SS regions (**E**) and compared with TNF-α treatment, which was set to 100%. Data are presented as mean ± SEM, dots in B–E represent values from individual experiments, *n* = 6–7, analyzed by one-way ANOVA followed by Dunnett’s multiple comparison (**F**) Exemplary microscopic images show monocytes (arrow) adhered to the endothelial cell layer (bar represents 100 µm). * *p* < 0.05, ** *p* < 0.01, *** *p* < 0.001, **** *p* < 0.0001.

The involvement of MAP kinases in SS-induced endothelial dysfunction was investigated using dynamic flow experiments (Figure 1A), followed by flow cytometric analyses to quantify levels of phosphorylated kinases. Compared with HUVECs under static conditions, exposure to SS increased phosphorylated JNK1/2 (*p* = 0.0460), as well as additional inflammatory stimulation with TNF-α (*p* = 0.0021) (Figure 1B). 

Higher levels of phosphorylated ERK (Figure 1C) were observed following TNF-α stimulation under static conditions (*p* = 0.0229), while a tendency to increased ERK phosphorylation was detected by combined inflammatory and mechanical stimulation (*p* = 0.06). No significant changes were found for phospho-p38K.

To investigate if JNK1/2 and ERK, which were induced by combined inflammatory and mechanical stimuli, are involved in monocyte adhesion to the EC monolayer under SS conditions, HUVECs were incubated with TNF-α and specific inhibitors of the respective kinases, followed by perfusion with THP-1 monocytes. Adhesion levels were quantified in regions of laminar and of non-uniform SS (Figure 1A). Under laminar SS, only a slight increase in THP-1 cell adhesion was observed in TNF-α compared with control treated cells (Figure 1D, *p* = 0.0149), while no significant changes were observed following suppression of either JNK1/2 or ERK. Under non-uniform SS, JNK1/2 inhibition suppressed TNF-α-induced adhesion to the endothelium (*p* = 0.0008), while ERK inhibition did not affect monocyte adhesion (Figure 1E,F).

### 2.2. Inflammatory Mechanoactivation of JNK1/2 Was Associated with the Induction of Cx43 and Caspase 3

**Figure 2:**
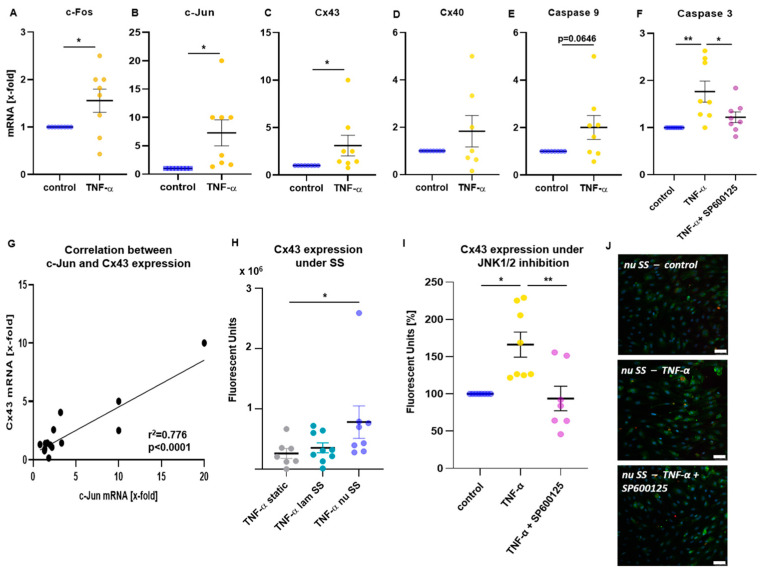
JNK activation in inflammatory- and mechano-stimulated ECs is associated with the induction of Cx43 and proapoptotic caspases. (**A**–**F**) HUVECs were seeded into bifurcation flow-through cell culture slides, creating regions of laminar and non-uniform SS. Cells were exposed to SS by application of flow for 20 h. For the last 2 h, cells were stimulated with TNF-α with or without addition of JNK1/2 inhibitor SP600125. Flow was stopped and RNA was isolated from HUVECs and reverse transcribed into cDNA to perform RT-qPCR. Transcription levels are illustrated as x-fold mRNA compared with control treatment, which was set to 100%. Under SS conditions, TNF-α induced expression of JNK-associated transcription factors (**A**,**B**), of gap junction protein Cx43 (**C**) and the proapoptotic proteases caspase 9 (**E**) and caspase 3 (**F**), the latest was suppressed by JNK1/2 inhibition. No significant changes were observed for Cx40 (**D**). Data are presented as mean ± SEM, *n* = 8, analyzed by unpaired t-test (**A**–**E**) or one-way ANOVA followed by Tukey’s multiple comparison (**F**). (**G**) Linear regression analyses show a correlation of c-Jun and Cx43 transcription levels. *n* = 15 (**H**–**J**) Flow experiments were performed as described and protein expression of Cx43 was quantified using immunofluorescence. (**H**) Cx43 protein expression was higher in TNF-α treated HUVECs exposed to non-uniform SS compared to static conditions. (**I**) Increase in Cx43 protein expression in non-uniform SS-exposed HUVECs following TNF-α stimulation was suppressed by JNK1/2 inhibition. Data are presented as mean ± SEM, dots represent values from individual experiments, *n* = 7–9, analyzed by Kruskal–Wallis test followed by Dunn’s multiple comparison. (**J**) Exemplary microscopic images of Cx43 expression under non-uniform SS are shown (DAPI = blue, Cx43 = green, Cx40 = red; bar represents 50 µm). * *p* < 0.05, ** *p* < 0.01.

RT-qPCR demonstrated an increased expression of JNK-associated signaling molecules by combined mechanical (exposure to laminar and non-uniform SS) and inflammatory stimulation in HUVECs. c-Fos mRNA levels showed a slight increase (Figure 2A, *p* = 0.0395), while transcription of c-Jun, another JNK downstream transcription factor, was also enhanced in mechanoactivated TNF-α stimulated HUVECs compared with the control (Figure 2B, *p* = 0.0158). Interestingly, we also observed an induction of the gap junction protein Cx43 (Figure 2C, *p* = 0.0422), while mRNA levels of Cx40 were not changed (Figure 2D). Apart from that, transcription of the proapoptotic cleaving enzyme, caspase 3, was increased by TNF-α stimulation (Figure 2F, *p* = 0.0032), but this effect disappeared when the JNK1/2 inhibitor was present (*p* = 0.0370 vs. TNF-α, *p* = n.s. vs. control), indicating an apoptotic response to the combined inflammatory and mechanical stimulation mediated by JNK1/2. Transcription of caspase 9 was slightly increased in TNF-α stimulated cells (Figure 2E, *p* = 0.0646). No significant changes were observed for IL-18, IL-1β and the Fas cell surface death receptor. Simple linear regression analyses of transcription levels showed a significant association of Cx43 with c-Jun (Figure 2G, r^2^ = 0.776, *p* < 0.0001).

To further investigate this correlation, Cx43 expression in mechanoactivated HUVECs was analyzed by immunofluorescence staining. Comparing static conditions to different shear stress patterns, expression of Cx43 was significantly higher in HUVECs exposed to non-uniform SS (Figure 2H, *p* = 0.0359 vs. static), while it was almost unchanged under laminar SS. In non-uniform SS regions, an induction of Cx43 expression was observed following TNF-α stimulation compared with control (Figure 2I,J, *p* = 0.0134), supporting the transcription results. Incubation with JNK1/2 inhibitor suppressed TNF-α-induced Cx43 expression (Figure 2I,J, *p* = 0.0038 vs. TNF-α; *p* = n.s. vs. control), suggesting that the induction of Cx43 was mediated by JNK activation.

### 2.3. Time-Dependent Cx43 Induction by Mechanical Stimulation

**Figure 3:**
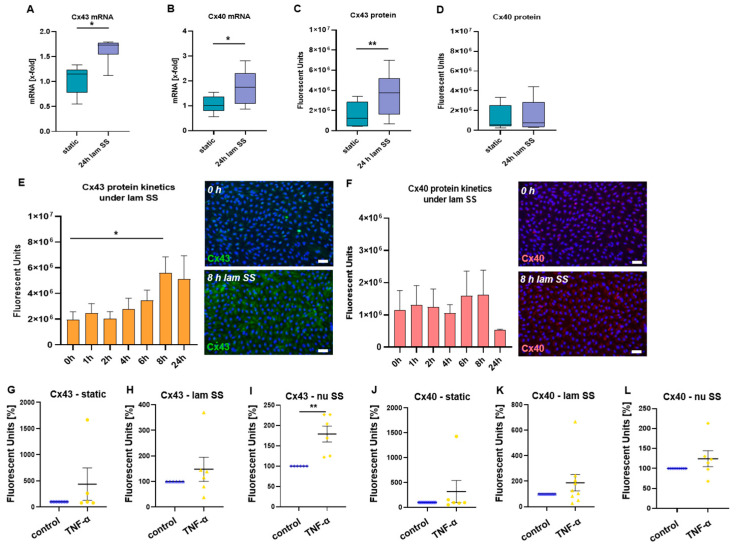
Mechanic and inflammatory induction of Cx expression in HUVECs. HUVECs were seeded into laminar (**A**–**D**) or bifurcation flow-through cell culture slides (**E**,**F**) and exposed to SS by application of flow for 0–24 h. (**A**,**B**) RNA was isolated from HUVECs following 24 h flow or static conditions for RT-qPCR, showing higher transcription levels of both Cx43 (**A**) and Cx40 (**B**) following laminar SS compared with static conditions (*n* = 6). (**C**,**D**) After 24 h of flow or static conditions, HUVECs were stained for Cx43 and Cx40 by immunofluorescence (*n* = 9). Cx43 expression was significantly higher under laminar SS compared with static conditions (**C**), while no significant changes were observed for Cx40 (**D**). Data are presented using box and whisker plot, showing median, the interquartile range and minimum and maximum values. Wilcoxon matched-pairs signed rank test (**A**,**D**) or paired t test (**B**,**C**) was used for analysis. (**E**,**F**) At different time points (0 h, 1 h, 2 h, 4 h, 6 h, 8 h and 24 h of flow) HUVECs were stained for Cx43 and Cx40 by immunofluorescence (*n* = 3–4). After 8 h exposure to laminar SS significantly higher expression levels of Cx43 were observed compared with 0 h (**E**), while no significant differences were observed for Cx40 (**F**). Data are presented as mean ± SEM. Unpaired t-test was used to analyze the different time points compared with 0 h. Exemplary microscopic images of immunofluorescent stained HUVECs are shown on the right (Cx43 = green, Cx40 = red, DAPI = blue, scale bar = 100 µm). (**G**–**L**) Impact of inflammatory stimulation on Cx expression in HUVECs exposed to static or flow conditions was analyzed by immunofluorescent stainings. Stimulation with TNF-α for 2 h had no effect on Cx43 and Cx40 expression under static conditions (**G**,**J**) or under laminar SS conditions (**H**,**K**). HUVECs exposed to non-uniform SS exerted higher Cx43 expression following TNF-α stimulation (**I**), while Cx40 expression levels were unchanged (**L**). Data are presented as mean ± SEM, dots represent values from individual experiments, *n* = 5–9, unpaired *t*-test was used for analysis. * *p* < 0.05, ** *p* < 0.01.

RT-qPCR demonstrated that mRNA levels of Cx43 (Figure 3A, *p* = 0.0313) and Cx40 (Figure 3B, *p* = 0.0386) were increased by exposure to 24 h laminar flow. At protein levels, 24 h flow increased Cx43 (Figure 3C, *p* = 0.0012), but no changes were observed in Cx40 (Figure 3D).

HUVECs were exposed to SS up to 24 h and time-dependent induction of Cx40 and Cx43 protein was investigated. Cx43 protein expression increased in a time-dependent manner and the increase was significant after 8 h of laminar SS (Figure 3E, *p* = 0.0420) while Cx40 protein expression did not change during 24 h of flow (Figure 3F).

### 2.4. Inflammatory Stimulation Acts as Booster of Cx43 Expression in Mechanoactivated HUVECs

To analyze if Cx expression is also induced by inflammatory stimulation, HUVECs were stimulated with TNF-α under static or flow conditions. While no significant changes in Cx43 were observed under static conditions (Figure 3G) and in regions of laminar SS (Figure 3H), it was significantly upregulated in HUVECs exposed to non-uniform SS (Figure 3I, *p* = 0.0024). Cx40 expression levels were not significantly affected by TNF-α, neither under static nor under flow conditions (Figure 3J–L).

### 2.5. Cx43 Is Involved in Proatherogenic Cell Responses in HUVECs Exposed to Non-Uniform SS

**Figure 4:**
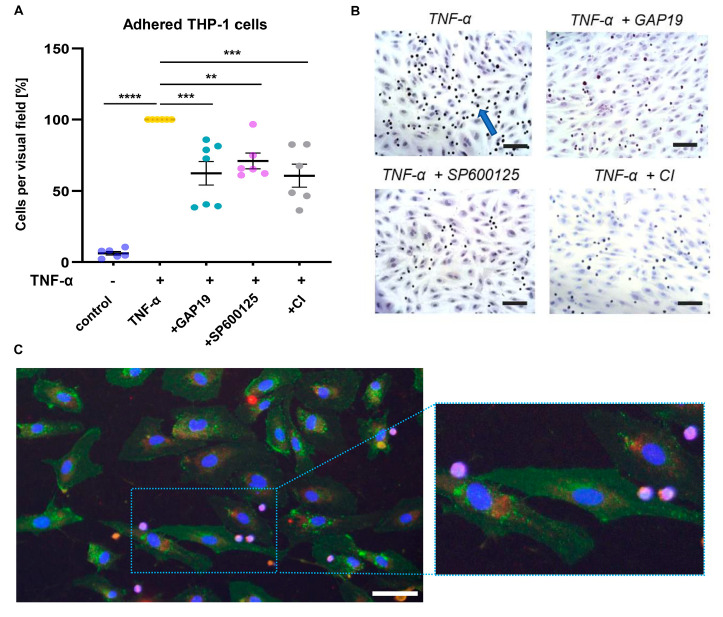
Cx43 and caspase 3 induction are involved in proatherogenic cell responses of HUVECs exposed to non-uniform SS. Cell adhesion assay was performed following 20 h of SS exposure. After 18 h of perfusion with medium, HUVECs were perfused with medium, TNF-α or TNF-α together with an inhibitor of Cx43 (GAP19) or of JNK1/2 (SP600125) or a caspase inhibitor (CI) for 2 h, followed by 1 h of perfusion with THP-1 monocytes. Cells were stained and microscopic images in regions of laminar SS (6 per slide) and non-uniform SS (8 per slide) were obtained. Mean numbers of adherent THP-1 cells per visual field were assessed in laminar and non-uniform SS regions (**A**) and compared to TNF-α treatment, which was set to 100%. Comparable to SP600125, incubation with GAP19 or CI together with TNF-α resulted in lower adhesion of THP-1 cells to the HUVEC monolayer than sole TNF-α. Data are presented as mean ± SEM, dots represent values from individual experiments, *n* = 6–7, analyzed by one-way ANOVA followed by Sidak’s multiple comparison. (**B**) Shown are exemplary microscopic images of monocytes (blue arrow) adhered to the endothelial cell monolayer under nun-uniform SS. Scale bar = 100 µm. (**C**) Immunofluorescent staining of Cx43 and Cx40 was performed following adhesion assay and fluorescence microscopic images were obtained in regions of non-uniform SS. Exemplary images show THP-1 monocytes (violet), which have adhered to the endothelium (DAPI = blue, Cx43 = green, Cx40 = red; bar represents 50 µm). ** *p* < 0.01, *** *p* < 0.001, **** *p* < 0.0001.

Using specific inhibitors, the role of Cx43 and caspase 3 in proatherogenic monocyte adhesion was investigated. Inhibition of Cx43 did not affect monocyte adhesion under laminar SS. In contrast, under non-uniform SS, monocyte adhesion was significantly reduced by both GAP19 treatment (*p* = 0.0005) and caspase-inhibition (*p* = 0.0004), in similar extent compared to JNK1/2 inhibition (Figure 4A,B, *p* = 0.0095).

Figure 4C shows an exemplary microscopic image of immunofluorescent-stained HUVECs following exposure to non-uniform SS and THP-1 cell adhesion. While only a weak expression of Cx40 (red staining) was verified, HUVECs exerted a pronounced expression of Cx43 (green staining), which was observed both intercellular between adjacent cells and intracellular, showing vesicular carriers for the transport of Cx43 to the plasma membrane. Adhered THP-1 monocytes were visualized by violet staining.

### 2.6. Expression of TNF-α and Cx43 in Human Carotid Plaque Specimens

Inflammatory gene expression was measured in endarterectomized human carotid plaques from patients with severe stenosis. Clinical data of the enrolled patient cohorts (*n* = 20 patients per cohort) are listed in Table 1. Significant changes were observed for fibrous cap (FC) thickness at the thinnest site, which was thicker in patients of the stable compared with the vulnerable cohort (*p* < 0.0001, analyzed by the Mann–Whitney test). Lipid core area of the plaque indeed was significantly smaller in patients of the stable cohort (*p* < 0.0001, analyzed by the Mann–Whitney test).

**Figure 5:**
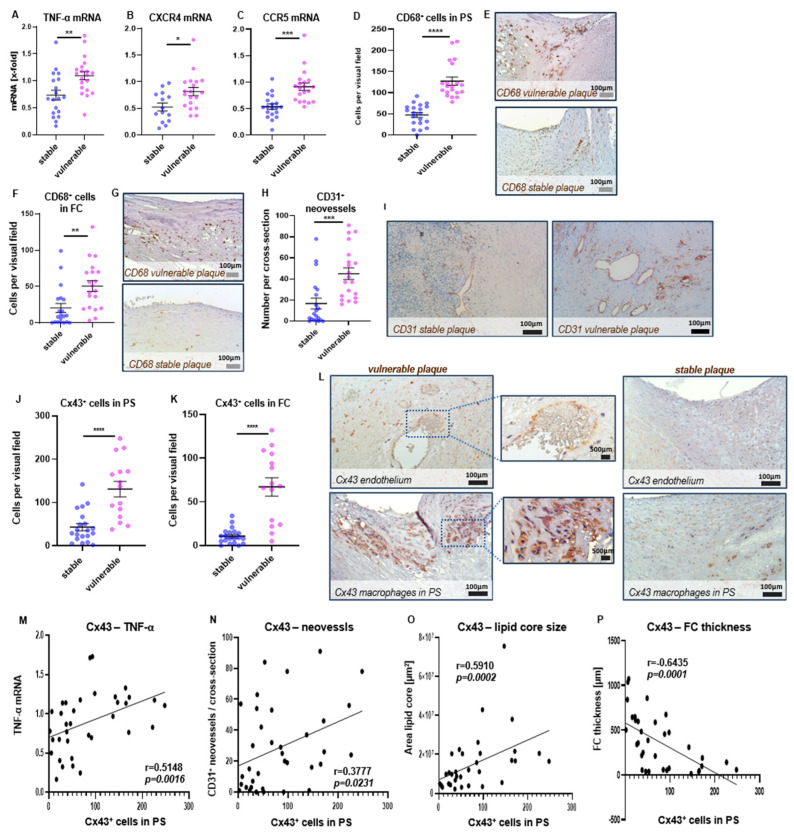
Role of Cx43 in clinical manifestation. (**A**–**C**) Transcription levels of TNF-α (**A**) and the chemokine receptors CXCR4 (**B**) and CCR5 (**C**) in stable and vulnerable plaques, *n* = 14–20. (**D**–**G**) Numbers of infiltrated CD68-expressing macrophages in the PS (**D**) and the FC (**F**), *n* = 20. Exemplary microscopic images of CD68-staining are shown of the PS I and the FC region (**G**). (**H**,**I**) Higher rate of CD31-stained neovessels in vulnerable compared with stable plaques (**H**), *n* = 20. Exemplary microscopic images, showing brown-stained endothelial cells of neovessels (scale bar = 100 µm) (**I**). **(J**,**K**) Increased numbers of Cx43-expressing cells in the PS region (**J**) and the FC (**K**) of vulnerable compared with stable plaques, *n* = 15–20. (**L**) Exemplary microscopic images show Cx43 expression by endothelial cells of neovessels (left upper image) and by macrophages in the inflammatory PS of a vulnerable plaque (right lower image), compared with Cx43-expressing cells in a stable plaque (right images). Data from gene plex assay and immunohistochemistry are presented as scatter dot plots showing mean ± SEM, dots represent values from individual experiments, unpaired t-test was used for analysis. (**M**–**P**) Spearman correlation analyses verify a correlation of Cx43^+^ cell counts with intraplaque TNF-α transcription, *n* = 35 (**M**), the rate of CD31^+^ neovessels, *n* = 36 (**N**), the mean area of the lipid core, *n* = 34 (**O**) and an inverse correlation with the FC thickness, *n* = 34 (**P**). * *p* < 0.05, ** *p* < 0.01, *** *p* < 0.001, **** *p* < 0.0001.

In vulnerable plaques, significantly increased transcription levels were observed in TNF-α (Figure 5A, *p* = 0.0045) and the two chemokine receptors CXCR4 (Figure 5B, *p* = 0.0136) and CCR5 (Figure 5C, *p* = 0.0001) compared with stable plaques. Transcription of CXCR3 was also slightly enhanced in vulnerable lesions, without reaching statistical significance (*p* = 0.1634), while no differences were detectable for IL-10. Apart from that, vulnerable plaques had an increased infiltration of CD68^+^ macrophages compared with stable plaques in both the plaque shoulder (PS) (Figure 5D,E, *p* < 0.0001) and FC region (Figure 5F,G, *p* = 0.003). Immunohistochemical analyses demonstrated enhanced intraplaque neovascularization in vulnerable plaques (Figure 5H,I, *p* = 0.0005), which was associated with higher numbers of Cx43-expressing cells in the PS (Figure 5J,L, *p* < 0.0001) and FC (Figure 5K, *p* < 0.0001). Cx43 was mainly expressed by endothelial cells, lining the vessel wall and intraplaque neovessels, and by macrophages (Figure 5L), whereas no expression was detected by CD3-expressing T cells. While in the lipid core and tunica media Cx43 was only weakly expressed, high amounts of Cx43-expressing cells were found in the shoulder regions of the plaque and also in the fibrous cap although less pronounced. Spearman correlation analyses revealed an association of Cx43^+^ cell counts in the PS region with intraplaque expression of TNF-α (r = 0.5148, *p* = 0.0016), CCR5 (r = 0.5526, *p* = 0.0006), local T cell and macrophage burden in the PS (T cells: r = 0.4485, *p* = 0.0061, macrophages r = 0.6864, *p* = 0.0001) and intraplaque neovessel formation (r = 0.3777, *p* = 0.0231) (Figure 5M,N and Table 2).

While Cx43^+^ cell counts did not correlate with the vessel lumen area, its association with the plaque/lumen ratio was found (Table 2, r = 0.4365, *p* = 0.0099). Cx43^+^ cell counts were significantly correlated with the mean lipid core area (Figure 5O, r = 0.591, *p* = 0.0002) and inversely correlated with the mean FC thickness (Figure 5P, r = −0.6435, *p* = 0.0001). Similar correlations were found in the FC (Table 2); however, in this plaque area no significant associations were observed with macrophage and T cell burden, nor with the amount of neovessels.

## 3. Discussion

In the present study, we showed that proatherogenic SS induces mechanoactivation of JNK1/2 in human ECs, which was associated with an induction of the gap junction protein Cx43. We also demonstrated that Cx43 was expressed by macrophages and endothelial cells in human carotid plaques, with Cx43^+^ cell counts associated with plaque vulnerability.

Investigation of three MAP kinases showed that exposure of ECs to SS had no effect on p38K phosphorylation in the applied setting, while ERK was activated by inflammatory stimulation, and JNK1/2 by either mechanic or inflammatory stimuli. The observed mechanoactivation of JNK1/2 is in accordance with former studies that demonstrated cyclic strain- and SS-induced increase in kinase activity [23,35]. In contrast to the results from Azuma et al., we did not observe changes in p38K phosphorylation, which might be explained by its rapid and transient activation readily 5 min after flow exposure [35]. As cells were exposed to flow for at least 20 h in our setting, p38K may have returned to its initial levels.

The effects of hemodynamic conditions or combined inflammatory stimulation on JNK activation have only been poorly explored so far [36]. Surapisitchat et al. found an activation of JNK and ERK by TNF-α stimulation in HUVECs [37]. In their study, a pre-exposure to laminar SS suppressed the inflammatory activation of JNK, which was discussed as beneficial impact of atheroprotective laminar SS conditions. We also observed an increased activation of JNK by TNF-α stimulation, however, this increase was also found under SS conditions. Indeed, SS-induced JNK activation was further enhanced by additional TNF-α stimulation. Of note, ECs in our experiments were exposed to flow in y-shaped slides, creating regions of atheroprone non-uniform SS. Our findings suggest that JNK1/2 activity depends on the respective flow pattern and shows pronounced activation under atheroprone SS conditions. Increased levels of phospho-JNK1/2 by TNF-α stimulation under flow conditions, while only marginal effects were observed under static conditions, refers to a boosted kinase activation in ECs following combined exposure to mechanic and inflammatory stimuli. Thus, JNK activation might also underlie an accelerated atherosclerosis progression in patients suffering from chronic inflammatory diseases, such as rheumatologic disorders [38] or psoriasis [39].

In the present study, adhesion assays confirmed a functional role of JNK1/2 in proatherogenic cellular interactions, induced by a combination of SS and inflammatory stimulation. Monocyte adhesion was most pronounced when ECs were exposed to both non-uniform SS and TNF-α stimulation. We are the first to show that inhibition of JNK1/2 suppressed TNF-α induced monocyte adhesion in regions of atheroprone non-uniform SS, while inhibition of ERK did not affect cellular adhesion. These findings support the hypothesis of a pronounced JNK activation driving proatherogenic cellular interactions in response to mechanical and inflammatory stimulation.

Apart from that, we demonstrated that JNK mechanoactivation was associated with an induction of Cx43, a gap junction molecule that is involved in intercellular communication and cytoplasmic exchange of metabolites between adjacent cells [40]. Induction of Cx43 was demonstrated in our study under atheroprone SS conditions, which was further upregulated by inflammatory stimulation. Under static conditions or atheroprotective laminar SS, Cx43 expression following TNF-α treatment was unchanged. JNK activation-dependent Cx43 induction was demonstrated by inhibition of JNK1/2, which completely abolished TNF-α-induced Cx43 gene expression in ECs exposed to non-uniform SS. Additionally, Cx43 mRNA levels correlated with those of the JNK downstream transcription factor c-Jun, supporting that Cx43 expression depends on JNK activation. In prion disease, JNK was also reported to mediate an upregulation of Cx43 in the affected brain tissue, which was estimated as pathogenic mechanism accelerating disease progression [33].

Regarding its functional role, Cx43 enables the cytoplasmic exchange of metabolites between cells. Former studies have shown that Cx43 also contributes to the transfer of proapoptotic signals between adjacent cells, promoting cell apoptosis [41,42]. In our study, Cx43 induction was accompanied by increased transcription levels of caspase 3 and caspase 9. As these proteases mediate membrane blebbing, which is observed as the onset of apoptosis induction [43], our findings suggest an apoptotic response of ECs to the combined stimulation by SS and TNF-α. Apoptosis of vascular cells, such as ECs and smooth muscle cells, has been reported to contribute to atherosclerotic plaque progression and, especially, to plaque rupture [44,45].

Interestingly, the inhibition of caspase and Cx43 both significantly suppressed THP-1 cell adhesion to ECs under non-uniform SS in the performed monocyte adhesion assays. Caspase 3 has been reported to cleave the Rho-associated protein kinase ROCK1 into its active form [43], which is involved in endothelial dysfunction. In a recent study, ROCK1 was shown to promote monocyte-endothelial adhesion by inducing endothelial expression of VCAM-1 and ICAM-1 [46]. Thus, reduced THP-1 cell adhesion, which we observed in ECs treated with a caspase inhibitor, might arise from a suppressed ROCK1 activity. An association of caspase 3 activation with Cx43 expression has already been described both in vitro and in vivo [47,48]. In rat hepatocytes, the suppression of Cx43 by siRNA transfection led to a decreased expression of caspase 3 and the proapoptotic Bid protein [48]. In addition to hepatocytes, apoptosis induction via Cx43 was reported in HUVECs by Ma et al. In this study, Cx43 inhibitor GAP19 suppressed apoptosis induced by Cx43 overexpression, which the authors assumed to result from an attenuated oxidative stress response [42].

Thus, the performed hemodynamic in vitro experiments in our study indicate that a combination of SS and inflammatory stimulation induces a proapoptotic response that is characterized by the induction of the gap junction protein Cx43. Increased JNK-activation induces surface expression of Cx43, in turn promoting intercellular transfer of proapoptotic stimuli, which mediates apoptotic cell death. A former study described the regulation of apoptosis by Cx43 and JNK1/2 in mesothelioma cells and found a direct interaction of Cx43 and JNK1/2 with Bax, an apoptosis-inducing protein [49]. The colocalization of phosphorylated JNK1/2 and Bax was only observed in cells expressing Cx43. Moreover, JNK1/2 activation as response to sunitinib treatment only appeared in the presence of Cx43, which mediated apoptosis via its interaction with Bax and JNK1/2 [49].

The investigation of human plaque specimens, obtained from patients with severe stenosis of the carotid artery, emphasize the role of TNF-α and Cx43 in atherogenesis. Hence, we provide novel evidence that Cx43 is expressed in human carotid plaques and that Cx43^+^ cell counts are associated with diverse markers of plaque vulnerability. Accordingly, a significant correlation with the degree of neovascularization and the size of the lipid core was observed, while a marker of plaque stability—FC thickness—was inversely correlated with Cx43^+^ cell counts. High amounts of Cx43-expressing cells were found in the plaque shoulder regions and in endothelial cells, bordering the vessel lumen and intraplaque neovessels. Since vulnerable plaques with higher Cx43^+^ cell counts compared with stable lesions also showed increased local macrophage and T cell infiltration, Cx43 seems to be involved in vascular inflammation. Confirming evidence came from transcription analyses showing an association of Cx43^+^ cell counts with intraplaque TNF-α and CCR5 transcription. CCR5 a chemokine receptor that has been reported to contribute to leukocyte recruitment and thus further progression of local inflammation [50,51]. Hence, mechanoactivation of Cx43 seems to be involved in human plaque progression.

The study has some limitations. First, we did not perform animal experiments functionally to confirm the involvement of JNK1/2 and Cx43 in SS induced proatherogenic mechanotransduction. However, we demonstrated that Cx43 is expressed by macrophages and endothelial cells in human carotid plaques. Apart from that, flow cytometry was performed to quantify phosphorylated levels of MAPKs using y-shaped μ-slides that contain both laminar and bifurcated regions. Accordingly, a comparison between SS and static conditions was possible, while we could not compare different local SS conditions.

## 4. Materials and Methods

### 4.1. Patients

#### Ethics Statement

The protocol of collecting human materials were approved by the institutional ethic committee of the university hospital Erlangen-Nürnberg and performed in concordance with the Helsinki declaration. All patients gave written informed consent to participate to the study before donating the materials.

### 4.2. Isolation of Human Umbilical Vein ECs (HUVECs) from Umbilical Cord

To investigate the impact of mechano- and inflammatory stimulation on endothelial activation in vitro, we performed dynamic flow experiments to analyze phosphorylation levels of MAPKs in human ECs following TNF-α stimulation under SS.

HUVECs were freshly isolated from umbilical cord veins as described in prior studies [52]. Briefly, the vein was flushed with saline several times, whereupon it was incubated with PBS (Sigma-Aldrich, Taufkirchen, Germany) containing a digestion enzyme (Dispase^®^, Cat. No. 17105041, Thermofisher, Waltham, MA, USA). Cells were collected and centrifuged for 10 min at 1000 rpm and the pellet was resuspended in 10 mL of EC Growth Medium (ECGM, PromoCell, Heidelberg, Germany). Cells were seeded into cell culture flasks and incubated at 37 °C with 7.5% CO_2_. When 80% of confluence was reached, cells were detached with accutase and used for experiments. If there was any doubt about the purity of the culture, i.e., contamination with vascular smooth muscle cells, the culture was discarded.

### 4.3. Analysis of Endothelial Mechanoactivation by Dynamic Flow Experiments

The role of different signaling kinases in SS-induced endothelial dysfunction was investigated using dynamic flow experiments. HUVECs (0.7 × 10^6^ cells/mL) were seeded into bifurcation flow-through cell culture slides (y-shaped μ-slides, Ibidi, Planegg, Germany). When confluence was reached, HUVECs were perfused with ECGM for 20 h at a flow rate of 9.6 mL/min, which creates regions of laminar SS (10.2–10.8 dyne/cm^2^) and regions of non-uniform SS, characterized by a SS gradient, ranging from ~0.5 dyne/cm^2^ to ~6.3 dyne/cm^2^ [53].

Subsequently, the following analyses were performed to explore the involvement of MAP kinases in endothelial mechanoactivation and downstream induced gene expression.

### 4.4. Flow Cytometric Analysis of Endothelial Mechanoactivation

As MAP kinases are activated by phosphorylation, intracellular staining of mechanoactivated HUVECs were performed to quantify phosphorylated kinases by flow cytometric analyses. For this purpose, HUVECs were perfused with TNF-α (2.5 ng/mL, Miltenyi Biotec, Bergisch Gladbach, Germany), or sole medium as control for the last 2 h of flow. Thereupon, HUVECs were collected following 5 min of incubation with accutase (Thermofisher) and washed with ECGM. Intracellular staining was performed according to the manufacturer’s instructions (BDTM Phosflow, BD Biosciences, Heidelberg, Germany) to quantify phosphorylated levels of JNK1/2 (PE Mouse anti-JNK (pT183/pY185), Cat. No. 562480), ERK (PE Mouse anti-ERK1/2 (pT202/pY204), Cat. No. 612566) and p38K (Alexa Fluor^®^ 488 Mouse anti-p38 MAPK (pT180/pY182), Cat. No. 612590). Stained HUVECs (10,000 cells per tube) were measured on FACS VERSE (BD Biosciences) and mean fluorescence intensity (MFI) of phosphorylated kinases was assessed using FACSuite™ software (BD Biosciences).

### 4.5. Immunofluorescent Staining

To investigate SS-induced protein expression, immunofluorescent staining was performed in both laminar and y-shaped μ-slides. HUVECs were fixed with 4% paraformaldehyde (Alfa Aesar, Karlsruhe, Germany) in PBS for 10 min. Fixed cells were permeabilized with 0.2% Triton-X 100 (Merck, Darmstadt, Germany) in PBS for 10 min and then blocked with 5% horse serum (Gibco, Eggenstein, Germany) in PBS for 30 min. Thereafter, cells were incubated with polyclonal rabbit anti-connexin 40 antibody (1:200, Cat. No. 36-4900, Invitrogen, Carlsbad, CA, USA) and monoclonal mouse anti-connexin 43 antibody (1:300, Cat. No. 13-8300, Invitrogen) at room temperature for 1 h, followed by incubation with Alexa fluor 555-conjugated anti-rabbit secondary antibody and 488-conjugated anti-mouse secondary antibody (1:500, Molecular Probes, Karlsruhe, Germany) for 45 min. Afterwards, cells were mounted with Fluoroshield™ mounting medium (Sigma-Aldrich). Under a fluorescence microscope (Olympus, Hamburg, Germany) 6 images in areas of laminar SS and 8 images in areas of non-uniform SS (Figure 1A) were captured at ×200 magnification using a digital camera (DS-FI1C, Nikon, Düsseldorf, Germany) and NIS Elements^®^ 4.30 software (Nikon). To quantify the fluorescence density, corrected total cell fluorescence (CTCF) was determined using Image J 1.52 software (National Institutes of health, Bethesda, MD, USA). CTCF was calculated using the following equation; CTCF = integrated density − (area of selected cell × mean fluorescence of background readings) [54]. For all images, a threshold was set to define the positive fluorescence signal intensity.

### 4.6. Adhesion Assay

Endothelial mechanoactivation is characterized by an increased adhesiveness of the EC layer, which can be analyzed by measuring the adhesion rate of monocytic THP-1 cells to ECs under SS conditions. To investigate the involvement of MAPKs and the MAPK-induced signaling cascade in SS-mediated cell adhesion, dynamic flow experiments were performed as described above. When TNF-α was applied, JNK1/2 inhibitor SP600125, or ERK inhibitor PD98059 (Sigma-Aldrich) were simultaneously added. Thereupon, ECs were perfused with THP1-monocytes (American Type Culture Collection, ATCC-No. TIB-202, 7.5 × 106 cells per slide) for 1 h of flow. After that, perfusion was stopped and slides were washed twice. Adherent cells were fixed by 4% paraformaldehyde and stained with hematoxylin and eosin. Microscopic images were obtained at ×200 magnification in regions of laminar SS (6 per slide) and non-uniform SS (8 per slide) using an inverted microscope (Olympus) with a mounted CCD camera (Nikon). Adherent THP-1 cells were counted and expressed relative to those of TNF-α treatment.

In an additional setting, Cx43 inhibitor GAP19 (Cat. No. 5353, Tocris, Wiesbaden-Nordenstadt, Germany), caspase-inhibitor (SCP0095, Sigma-Aldrich) were used to evaluate the impact of the gap junction molecule Cx43 and of proapoptotic cleaving enzymes (caspases) on monocytic cell adhesion.

### 4.7. Quantitative Real-Time PCR

HUVECs were harvested from bifurcation slides following 20 h of flow or static conditions and RNA was extracted from cells using a commercial kit (Qiagen, Hilden, Germany). RNA was reverse transcribed into cDNA following the manufacturer’s instructions (QuantiTect^®^ Reverse Transcriptase Kit, Qiagen). Quantitative real-time PCR (RT-qPCR) was performed using iQTM Syber^®^ Green Supermix (Bio-Rad Laboratories, Inc., Hercules, CA, USA) and QuantiTect^®^ Primer Assays (Qiagen) as listed in Table 3. As housekeeping genes, HPRT1 and YWHAZ were used. Transcription levels were normalized to the samples treated with TNF-α, which were set as 100%.

### 4.8. Collection of Carotid Plaque Specimens of Patients Undergoing Endarterectomy

To analyze local plaque inflammation in patients with different plaque stages, carotid specimens were collected from 40 patients with carotid artery disease. Degree of luminal stenosis of the internal carotid artery was measured by preoperative duplex scanning, magnetic resonance imaging, or angiography of the carotid arteries. Endarterectomy was performed if stenosis was more than 70% for symptomatic patients and more than 80% for asymptomatic patients. Detailed clinical data of the patients enrolled in the study are shown in Table 1.

### 4.9. Histological Analysis of Plaque Specimens

The removed carotid specimens were formalin-fixed, decalcified and further processed as described elsewhere [52]. Cross-sections with a thickness of 4 µm were prepared (3 sections per plaque, 100 µm apart from each other) for trichrome and hematoxylin-eosin staining to classify plaques into vulnerable and stable lesions, based on established parameters, comprising i.e., FC thickness, lipid core size, and hemorrhages [55].

Additionally, cross-sections were immunohistochemically stained for CD68 (mouse-anti-human, ready to use, DAKO, Hamburg, Germany) to detect plaque migrated macrophages, CD3 (mouse-anti-human, DAKO, 1:50) to detect T cells and Cx43 (mouse-anti-human, Invitrogen, 1:100) using CSA-Kit (DAKO), according to the manufacturer’s instructions. CD31 (mouse-anti-human, DAKO, ready to use) was stained to assess intraplaque neovascularization. Briefly, after deparaffinization in xylene and rehydration in decreasing concentrations of ethanol, demasking of antigens was performed by boiling sections in citrate buffer (pH = 6.0). Endogenous peroxidase was blocked, whereupon sections were incubated with protein block to suppress non-specific binding. After incubation with the primary antibodies or the corresponding negative control, sections were incubated with secondary biotinylated link antibody (rabbit-anti-mouse, DAKO, undiluted). Streptavidin-Biotin-Peroxidase complex was incubated, followed by biotinylated tyramide for signal amplification and streptavidin peroxidase incubation. Diaminobenzidine (DAB) was used as chromogen and nuclei were counterstained by hematoxylin.

Microphotographic images of the FC and the PS region were obtained at ×150 magnification. Number of infiltrated macrophages and T cells and of Cx43-expressing cells were manually counted in a representative area of 0.25 mm^2^ for each plaque region (FC and PS) in a blinded manner, using NIS Elements^®^ 4.30 software (Nikon). Number of CD31-expressing neovasculature was counted per plaque cross-section.

### 4.10. Analysis of Cytokine Expression in Human Carotid Plaques

To isolate RNA from the plaque specimens, cross-sections with a thickness of 4 µm were obtained and dried for 1 h at 60 °C. Gene expression assay was performed as previously described [56] using a commercially available kit (QuantiGeneTM plex assay, Invitrogen) with specified fluorescent-labeled probe sets for hybridization of TNF-α, IL-10, CXCR3, CXCR4, and CCR5, and the two housekeeping genes ubiquitin and HPRT1. Briefly, slides were deparaffinized in Xylol, followed by two 5-min incubation in 100% ethanol. Tissue was scraped off from the slides and lysed overnight in a supplied buffer containing proteinase K. Tissue homogenates were pipetted in duplicates into a hybridization plate and a master mix (containing capture beads, proteinase K, blocking reagent, lysis mixture, nuclease-free water and the selected probe sets) was added.

RNA hybridization was performed overnight by shaking the plate at 600 rpm in a hybridization oven at 54 °C. On the next day, amplification and washing steps were performed according to the manufacturer’s instructions. The fluorescent intensity was measured on a MAGPIX multiplex reader (Bio-Rad Laboratories). Obtained data was normalized to the housekeeping genes and expressed as x-fold of a selected sample which was defined as internal reference probe using Bio Plex Manager 6.1 Software (Bio-Rad Laboratories).

### 4.11. Statistics

Statistical analyses were performed using GraphPad Prism 8 (GraphPad Software, San Diego, CA, USA). Kolmogorov-Smirnov test served to analyze data for normal distribution. Two groups were statistically compared using Student’s *t*-test for normally distributed data or Mann-Whitney-test as non-parametric test. One-way ANOVA with the respective post hoc tests was used to compare multiple groups. To analyze non-random associations between two variables, Fisher’s exact test was used. Correlation between different parameters was analyzed using simple linear regression or Spearman correlation analyses. Data were expressed as mean ± SEM unless stated otherwise. *p* < 0.05 was considered statistically significant.

## 5. Conclusions

Taken together, the findings of our study suggest that JNK1/2 mechanoactivation in human ECs is involved in a proatherogenic cell response to non-uniform SS, which is observed in curved vessel segments or bifurcations, where atherosclerotic plaque preferentially develop. A boosted JNK activation by additional proinflammatory stimuli may accelerate atherosclerotic plaque development. JNK1/2 mediated mechanoinduction of Cx43 resulted in the enhancement of proinflammatory cell adhesion to ECs at areas exposed to non-uniform SS. A pronounced expression of the gap junction protein Cx43 in human carotid plaques and its correlation with markers of plaque vulnerability suggests the involvement of Cx43 in human plaque progression.

## Figures and Tables

**Table 1:** Clinical data of the enrolled patient cohorts.

Parameter	Cohort 1 = Stable Plaque	Cohort 2 = Vulnerable Plaque	*p*-Value
Age	73.1 ± 2.1	76.9 ± 1.6	n.s.
Sex, male, *n* (%)	13 (65)	15 (75)	n.s.
BMI	27.1 ± 1.1	28.0 ± 1.1	n.s.
Ischemic symptoms, *n* (%)	2 (10)	6 (30)	n.s.
Diabetes, *n* (%)	3 (15)	8 (40)	n.s.
Smoking, *n* (%)	7 (35)	9 (45)	n.s.
Hyperlipidemia, *n* (%)	11 (55)	12 (60)	n.s.
Hypertension, *n* (%)	17 (85)	17 (85)	n.s.
CRP (mg/dL)	1.5 ± 0.5	1.0 ± 0.2	n.s.
Creatinine (mg/dL)	1.2 ± 0.2	1.0 ± 0.1	n.s.
LDL-cholesterol (mg/dL)	127.7 ± 12.7	128.7 ± 6.0	n.s.
FC thinnest site (µm)	421 ± 50	84 ± 23	<0.0001
Lipid core area (mm^2^)	7.8 ± 1.4	23.9 ± 3.9	<0.0001

n.s. = not significant, BMI = body mass index, CRP = C-reactive protein, FC = fibrous cap, LDL = low-density lipoprotein.

**Table 2:** Spearman correlation coefficient r of human plaque parameters.

Parameter	Cx43^+^ Cells PS r	Cx43^+^ Cells PS *p*-Value	Cx43^+^ Cells FC r	Cx43^+^ Cells FC *p*-Value
TNF-α mRNA	0.5148	0.0016	0.5405	0.0010
CCR5 mRNA	0.5526	0.0006	0.5483	0.0008
CXCR4 mRNA	0.3287	n.s.	0.3101	n.s.
CD68^+^ cells PS/FC	0.6864	0.0001	0.2037	n.s.
CD3^+^ T cells PS/FC	0.4485	0.0061	0.2917	n.s.
CD31^+^ neovessels	0.3777	0.0231	0.2717	n.s.
Ratio Plaque/Lumen	0.4365	0.0099	0.2998	n.s.
Mean lipid core area	0.5910	0.0002	0.6665	<0.0001
FC (thinnest site)	−0.5235	0.0015	−0.5274	0.0016
Lumen area	−0.2173	n.s.	−0.0714	n.s.

FC = fibrous cap, PS = plaque shoulder, n.s. = not significant.

**Table 3:** Qiagen QuantiTect^®^ Primer Assays used for RTq-PCR.

Target Gene	Primer Assay	GeneGlobe ID
Cx40 (gap junction protein alpha 1)	Hs_GJA1_1_SG	QT00012684
Cx43 (gap junction protein alpha 5)	Hs_GJA5_va.1_SG	QT01001315
c-Fos (FBJ murine osteosarcoma viral oncogene homolog)	Hs_FOS_1_SG	QT00007070
c-Jun (jun proto-oncogene)	Hs_JUN_1_SG	QT00242956
IL-18 (Interleukin 18)	Hs_IL18_1_SG	QT00014560
IL-1 beta (Interleukin 1 beta)	Hs_IL1B_1_SG	QT00021385
Fas (Fas cell surface death receptor)	Hs_FAS_1_SG	QT00030618
Caspase 3 (apoptosis-related cysteine peptidase)	Hs_CASP3_1_SG	QT00023947
Caspase 9 (apoptosis-related cysteine peptidase)	Hs_CASP9_1_SG	QT00036267
HPRT1 (hypoxanthine phosphoribosyl-transferase 1)	Hs_HPRT1_1_SG	QT00059066
YWHAZ (tyrosine 3-monooxygenase)	Hs_YWHAZ_1_SG	QT00087962

## Data Availability

Data available on request from the authors.
